# The Dispensable Surplus Dairy Calf: Is This Issue a “Wicked Problem” and Where Do We Go From Here?

**DOI:** 10.3389/fvets.2021.660934

**Published:** 2021-04-14

**Authors:** Sarah E. Bolton, Marina A. G. von Keyserlingk

**Affiliations:** ^1^Animal Welfare Program, Faculty of Land and Food Systems, University of British Columbia, Vancouver, BC, Canada; ^2^Dairy Australia, Southbank, VIC, Australia; ^3^Faculty of Veterinary and Agricultural Sciences, The University of Melbourne, Melbourne, VIC, Australia

**Keywords:** animal welfare, dairy calves, participatory methodologies, ethics, complex problems

## Abstract

Surplus dairy calves consist of all dairy bull calves and any heifer calves not needed as replacements for the milking herd. The fate of these surplus calves varies by region; for example, in Australia and New Zealand they are often sold as “bobby” calves and slaughtered within the first weeks of life; whereas, in North America they are normally sold within the first weeks of life but reared for 16–18 weeks as veal or longer as dairy beef. Regardless of region, demand for these calves is often very low, driving down prices and in some cases leaving farmers with no alternative options other than on-farm euthanasia. The notion that dairy cows must give birth to produce milk and that the calves are immediately separated from the dam, many of which will end up immediately being sold as surplus calves, has become a topic of public concern. These concerns have increased given the growing number of pictures and stories in the media of on-farm euthanasia, dairy calves being transported at very young ages and frequently receiving sub-standard levels of care. In this paper we describe the status quo of this complex, value-laden issue that without transformative change is at great risk for continued criticism from the public. Moreover, despite many attempts at refinement of the existing approach (i.e., the pursuit of technical improvements), little has changed in terms of how these surplus dairy calves are managed and so we predict that on its own, this approach will likely fail in the long run. We then set out how the current surplus calf management practices could be viewed to fit the definition of a “wicked problem.” We conclude by calling for new research using participatory methodologies that include the voice of all stakeholders including the public, as a first step in identifying sustainable solutions that resonate with both society and the livestock industry. We briefly discuss three participatory methodologies that have successfully been used to develop sustainable solutions for other complex problems. Adoption of these types of methodologies has the potential to help position the dairy industry as a leader in sustainable food production.

## Introduction

The issue of surplus calves in dairy production has historically been limited to the fate of the male calf (1–3). However, the increasing use of sexed semen to strategically breed replacement females (4) combined with the growing demand for beef crossbreeding on the remainder of the herd (5), has resulted in an increasing proportion of these surplus calves being female. The current fate of most of these dispensable surplus calves is fraught with criticisms due in large part to a history of poor management, such as inadequate colostrum provision (6), transportation within a week of birth, young calves being sold through auction yards, and high rates of morbidity and mortality [see (3, 7)]. Given the increased concerns raised by critics regarding contentious practices in animal agriculture [i.e., see example of male chicks in Germany described by Brümmer et al. (8)], we predict an increasing awareness of potentially contentious issues being circulated through news reports and social media posts.

Citizens are increasingly expressing concern for the quality of life of farm animals (9). Without understanding societal values, food animal industries may implement improvements that are intended to improve animal welfare but are viewed as unacceptable to the public. For example, as described by Weary et al. (10), after years of public outcry over the use of confined housing for laying hens, millions of dollars, and years of research were spent on developing new “modified” cages that incorporated the latest collective scientific knowledge on social group size, space allowance and needs of the hens in these systems (11, 12). However, these “modified” systems failed to resonate with the key societal demand for cage-free systems; had the egg industry done the necessary consultation and reflection on these public values, the industry investment and scientific effort may have been more wisely devoted to improving cage-free rearing systems. To avoid similar missteps by the dairy industry, we suggest that future solutions must integrate the views of the public in developing approaches to address contentious practices, potentially contributing to the social license to farm.

The thoughts and ideas that are presented in this paper arose as a consequence of weekly online video discussions undertaken by the two authors who live on opposite sides of the world over a 10-month period, that began at the outset of the COVID-19 pandemic. In our weekly conversations, we discussed many unique challenges facing our respective dairy industries but quickly realized that regardless of where one lives, the fate of the surplus dairy calf is an ever-present challenge. Moreover, the majority of the available scientific literature suggests that most, if not all, research dedicated to surplus calves has focused on “technical issues” such as whether male dairy calves receive sufficient colostrum (6) or describing the *status quo* which includes most surplus calves either being transported off the farm at less than 1–2 weeks of age or euthanized at birth (13). Hence our discussions moved to focus on what alternative solutions could be found that would support a more socially sustainable dairy industry.

This paper summarizes these discussions into four parts beginning with a short description of the *status quo* of surplus dairy calf management and the case for change. In this section we have, given our respective locations, primarily used examples from Australia and Canada but when possible also included examples from other countries. We then argue that attempts to date to improve the welfare of surplus calves have been limited to technical solutions that have focused on refinement of existing practices and discuss why this approach may fail in the long run. We then explore whether the challenge of surplus dairy calf management may fit the definition of a wicked problem, before finally moving to describe how the use of participatory methodologies may assist with developing sustainable paths forward. We have also included real-world examples where these types of approaches have been used to effectively tackle wicked problems and discuss how research is needed on adapting these approaches so that they may be applied to the fate of the surplus dairy calf (and arguably other contentious issues).

## The Status Quo

In order to produce milk, cows must give birth to a calf (14) that, under natural circumstances, would suckle the cow until weaning occurs when calves are 7–9 months of age (15). In contrast, the majority of conventional dairy farms separate calves from the dam within 24 h of birth (16, 17). For the dairy industry to produce milk efficiently, farmers strive to achieve a yearly calving cycle; namely, every cow produces one calf every year. Considering replacement rates of lactating dairy herds (18), ~30–50% of the calves born on farms will be reared as replacement milking females while the remaining surplus female calves and all male calves must be managed through alternative pathways. In a study of calves sold at auction for veal operations in Quebec, Canada, 13% of calves sold were female (19), indicating that the issue of surplus dairy calves can no longer be confined to a focus on male calves alone.

Since the 1940s, genetic selection has seen the modern dairy cow become highly specialized, producing more milk from less inputs and improving overall efficiency (20). However, it appears that this selection for high milk production has been largely at the expense of beef production traits. In comparison to specialized beef breeds, many dairy breed offspring exhibit reduced average daily gains, lower dressing percentages and less desirable carcass conformation (21, 22), impacting their suitability for, and use in, profitable meat production systems.

As a result of their perceived lack of suitability for beef production, the majority of surplus dairy calves in Quebec and Ontario, Canada's major dairy provinces, enter the veal industry [see (23)] and are slaughtered when they are 16–18 weeks old (24), a management practice that has not changed dramatically in decades despite consumption rates of veal declining in North America; as of 2016 the annual veal consumption within Canada has dropped below 1 kg per person and to less than 100 g per person in the United States (25, 26). The continued reliance on the veal industry as a viable and sustainable market by the Canadian dairy industry and elsewhere must be questioned, particularly given that animal welfare and ethical concerns are the most commonly cited reasons for not consuming veal (27).

Concerns regarding the welfare of young surplus calves are not limited to North America. In Great Britain, the 0–3 months death rate at slaughterhouses for male dairy calves has increased from 17.4% in 2011 to 26.16% in 2018, in contrast to that of female dairy calves and beef calves of both sexes which has remained low (<0.5%) (28). In Australia, there is little in the way of established veal or dairy beef markets resulting in most surplus dairy calves entering the bobby calf market (29) where they are slaughtered within the first weeks of life (30).

The reduced suitability of dairy breed calves for beef production is also reflected in the value attributed to them, with Brown Swiss and Jersey calves attracting the lowest prices in a recent Canadian study, followed by calves with Holstein genetics, while cross-bred calves with beef genetics sold for higher prices (7). Similarly, Buczinski et al. (31) found that beef cross-bred calves sold through auction markets of Quebec had better sale prices than Holstein; whereas, colored dairy calves had lower sale characteristics than both Holstein and beef cross-bred calves. We speculate that the low inherent value of surplus calves motivates, at least in part, their sale at a young age. Wilson et al. (7) also report that Holstein dairy calves sold at auction were similar in body weight (~47 kg) to those of newborn female Holstein calves born in the same region in Canada (32) and elsewhere (33), suggesting that the majority of the Holstein calves in these studies were less than a week old when sold. It should be noted that in Canada, as of February 20, 2020, new federal regulations prohibit transporting calves with unhealed navels, and require that calves under 9 days of age be transported directly from farm to farm without going through an auction or assembly yard. The maximum trip length must be no longer than 12 h—shorter than typical trips for many surplus calves being transported in Canada which often exceeds 12 h and may be up to 48 h in duration (3). Unweaned calves aged 9 days or older can be sold at auction, but the total trip from dairy farm to calf grower cannot exceed 12 h except in specially equipped transport trailers (34). Similar regulations exist in Australia where, amongst other requirements, calves must not be transported before 5 days of age (unless consigned directly to a calf rearing facility), must be fit and healthy and fed within 6 h of transport with a maximum journey of 12 h [see (35)].

Given that the core business focus of most dairy farmers is on milk production, and that surplus calves are often of low value and in some cases are viewed as a “waste product” (36), it is not surprising that the standard of care provided to these calves is often lower than that afforded to arguably higher value replacement female calves. In a recent Canadian survey, 9% of farmers indicated that they did not always feed colostrum to male calves (a practice essential for managing the incidence of disease), and 17% did not provide the same quantity of feed to male calves as they did to heifer calves (23). This was further supported by the views of Canadian veterinarians in one study, where participants noted that if bull calves are “…*worth twenty bucks, they get fed, sort of”* and that “*they might not even really get colostrum”* (36). In the UK, male dairy calves were also found to have the highest on-farm mortality rates in the first three months of life when compared to female and beef breed calves (28). High rates of health abnormalities including diarrhea, dehydration, navel inflammation and low body condition have also been reported in calves sold at auction in Canada (7, 19) and upon arrival at milk-fed veal calf facilities (2, 37, 38). The most recent data from the US National Animal Health Monitoring System indicates the majority of the 42 operations surveyed sold their bull calves before weaning, with most doing so when the calves were less than 1 week old and about half of these were sold via an auction yard (6).

The transportation required to relocate surplus calves from the farm on which they are born to either a rearing facility or to slaughter also impacts their welfare. Calves are often transported within a week of life (29), including within a day of birth (3), with mortality of calves less than a week old increasing exponentially with distance traveled (39). Particularly worrisome is that the time spent during transport usually equates to time that they do not have access to milk; a fact that has been shown to directly impact their welfare (1). This notion that time off feed is a risk factor for mortality was acknowledged by a group of Canadian dairy industry experts who noted that young calves have limited body reserves to meet the demands of transport, which can have a duration of up to 48 h including a rest stop (3). These experts also noted that stress caused by handling can suppress immunity to disease [see also Burdick et al. (40)], that commingling of calves from different farms exposes them to new pathogens [see also Damiaans et al. (41)] and that calves do not always receive appropriate quantity and quality of feed and water while in the transport continuum (3).

Given the economic challenges associated with surplus calves, it is not surprising that in some instances they are euthanised on-farm shortly after birth (23, 29); a decision that in some cases likely arises as the farmers are forced to make the trade-off that the value attributed to a calf is less than the cost of rearing it, a fact likely exacerbated when there are minimum age requirements for transport. Decisions regarding euthanizing healthy calves shortly after birth is likely compounded in situations where farmers face a lack of access to housing facilities for these surplus calves (23). While the majority of Australian farmers euthanising calves do so with firearms (29), the use of blunt force trauma (euthanasia via a sharp blow from a solid object to the head) continues to be used by some, posing a significant risk of poor welfare outcomes resulting from issues with operator training and error (42). In one survey of Canadian farmers, an average of 19% of calves were euthanised at birth and of those respondents that euthanised calves, 34% reported using blunt force trauma, a practice that is not acceptable under both the Canadian Code on the Care and Handling of Dairy Cattle (23) and by the American Veterinary Medical Association see (43), and is also against Australian Dairy Farmers policy [see (44)]. Objectively, immediate and effective euthanasia following birth may be a preferable welfare option than experiencing standards of care that are common to surplus calves, such as long periods off feed, transportation or other known stressful conditions (e.g., cold) that can increase the risk of disease. Despite this, the killing of a newborn will not be accepted easily by the public due to ethical concerns; a point that will likely increase reputational risk to the industry. For example, the publishing of an undercover video taken on a dairy farm operating in Chile, with links to the New Zealand dairy industry, reporting that over 6,000 calves had been killed using blunt force trauma resulted in public outcry in New Zealand [described by (45)]. The voices of criticism following publication of the video were sufficient to enact changes in the New Zealand Animal Welfare Act (46) making it “*illegal to kill a calf by blunt force to the head, except in emergency circumstances.”* Clearly, the fact that the surplus calf is viewed as dispensable and killed immediately following birth in some regions of the world or allowed to live but given substandard care (at least relative to the female replacement heifers) is not socially sustainable and so alternative options must be explored.

### The Case for Change

The *status quo* of how surplus calves are cared for has, as argued above, primarily been brought about in part because these animals have an inherent low economic value and in part because the dairy industry is focused on the milk production aspects of their industry. However, the rising value attributed to the maintenance of public trust in the dairy industry has initiated discussions about the need to improve the way surplus calves are managed (45, 47). Commonly recognized challenges facing agriculture more broadly include a general public that is becoming increasingly disconnected from food production (48), combined with an increase in concern about how food is produced (49, 50).

When it comes to dairy farming, concerns about the welfare of animals are amongst the most commonly cited by the public (51). Indeed, the management of bobby calves has been rated as one of the most significant issues facing the Australian Dairy Industry and is recognized as a key barrier hindering the long term sustainability of the industry (52). There is a growing sensitivity globally that this issue must be addressed, exemplified by the views of a Canadian veterinarian who noted that “*if the public was more aware of what was going on there, it's not probably going to make good press”* (36). Unsurprisingly, when Australian study participants having little knowledge of the dairy industry were informed about the reason for the slaughtering of bobby calves, they responded with a high level of outrage and farm animal welfare standards were perceived as being inadequate (48). There is also some evidence in the media that the issue of surplus calves will likely be tied to cow calf separation (53, 54) which we predict will add additional complexity to this issue.

The increasing force of the social push-back by members of society regarding the management of surplus dairy calves has potential economic consequences, particularly in light of the rising interest in socially responsible finance (55), with some banks now promoting lending positions that exclude systems and processes that have negative impacts on animal welfare [see (56)]. It may also contribute to difficulties in attracting and retaining new entrants to the dairy sector, exemplified by the comment from a Canadian veterinarian: “*we see a lot of the younger generation that's coming on to the farm that seem to really want to push the calf welfare issue”* (36). Whether future economic pressures play a role in facilitating improved surplus calf management, particularly when considering the opportunities for increased revenue from beef, remains to be seen. Regardless, change is not easy as stated by some Canadian farmers who participated in a focus group study where they emphasized that money is necessary to make on-farm changes and meet the must-haves of farms in 20 years (57).

The case for shifting away from regarding the surplus calf as a waste product of dairy systems is not confined to social and economic pressures. Multiple studies have reported that beef from the dairy herd has a lower carbon footprint compared to beef from traditional beef herds (58–61), making this form of beef production potentially very attractive, particularly in the context of climate change. This potential advantage of dairy beef is attributed to emissions from the breeder cow being allocated between the various products. In the case of dairy beef production, the dairy cow produces milk, meat, and calves, with emissions allocated among all three products compared to the beef suckler cows which only produce meat and calves (60, 61). This explanation suggests that improving the uptake of beef from the dairy herd could lead to improved land use efficiency, which will be required in order to meet future increases in food production (62).

### Current Approaches to Achieving Change

There is little doubt that the dairy industry has some appetite for change, one only has to look at the structural changes that have occurred over the last 50 years (63). However, these changes have for the most part been driven by the pursuit for improved production efficiencies, such as increased milk production per cow through the adoption of improved genetics (64), scientific advances in ruminant nutrition [i.e., (65, 66)] and adoption of technologies to aid in health monitoring [see (67)]. When it comes to surplus calves, approaches to achieving change have largely focused on improving practices such as colostrum management (68), euthanasia practices (69) and transport standards (39), and increasing the adoption of technologies such as sex-sorted semen (4, 70).

Increased adoption of sex-sorted semen, which allows predetermination of calf sex with ~90% reliability (70), will affect surplus calf management as it provides for more targeted breeding of replacement females. Advantages of sexed semen can include accelerated rates of genetic gain in the female herd (71) and reduced dystocia rates due to smaller female calves, although potential reductions in fertility can reduce the financial benefits associated with implementation (70). Most notably, the combined use of sexed semen to produce the required number of replacement females with beef crossbreeding over the remainder of the herd has the potential to improve the value of surplus calves (72, 73). Indeed, recent evidence suggests that the feedlot performance, carcass quality and yield of crossbred Jersey calves sired by beef breeds was improved compared to purebred Jersey calves (74). Undeniably, a focus on improving the technical feasibility of more sustainable surplus calf management practices is a fundamental requirement to achieving change. However, despite the widespread availability of these technical advancements, the problem of surplus calf management persists, suggesting that this approach alone may be insufficient.

Unique marketing angles have also been suggested as an approach to improving surplus calf management by increasing the financial returns of beef from the dairy herd. This may provide gains in niche markets; however, Appleby (75) notes that “*it is not reasonable to expect consumers to take day-by-day responsibility for animal welfare at the point of sale, any more than they are expected to do so for other issues of concern to society, such as pollution.”* Whilst niche markets may offer a partial solution, it is unrealistic to expect this approach to act as a panacea.

Approaches to preserving trust in the dairy sector also faces a lack of consensus amongst stakeholders. The fact that many communities are increasingly disconnected from agriculture, has caused many within the industry to dismiss the general public as simply not knowledgeable (76). However, restricting the flow of information (often referred to as “ag-gag” laws) has been shown to be counterproductive, decreasing trust in farmers and leading to more negative perceptions of farm animal welfare standards (9).

Educating the public as a means to gain acceptance is another approach commonly argued by those within agriculture as a way of preserving trust [discussed by (36, 57)]. However, proponents of the education approach often fail to recognize that it will likely also highlight aspects that fail to resonate with societal values [e.g., zero grazing, cow calf separation reviewed by (10, 77, 78)]. This is compounded by the fact that animal welfare is often assessed by citizens not just in light of biological functioning, but also through the lenses of “naturalness” and affective states (i.e., the way the animals feel) (79).

Given that closing the doors or educating the public into understanding is unlikely to adequately address the threat of diminishing public trust in dairy production (45), how then should the industry proceed? Whilst technical solutions for improving surplus calf management are available and utilized to some extent, the persistence of the issue at a global level brings into question whether the problem must be viewed differently to those that are tackled solely through traditional scientific approaches targeted at refinement of existing practices.

### Why the Status Quo May Be a Wicked Problem?

Despite the refinement efforts made to date, there remain few, if any, dairying countries that do not experience some form of challenge when it comes managing surplus calves. In short, the issue is yet to be completely “solved,” despite our best efforts in research, development and extension.

The inherent division between the separate beef and dairy sectors present in many countries may play a role, at least to some degree, in hindering the development of sustainable solutions to the surplus dairy calf issue. Other challenges hindering progress may include commodity price volatility and inherent aversion to financial risk by many dairy producers (57), arguably resulting in current management practices continuing to place most emphasis on the path with least economic resistance. Possible differences in cultural attitudes to the perceived quality of dairy beef or veal both within the agricultural sector as well as amongst consumers may also play a role. Further, the concept of “barn blindness”—a lack of perception of problems on one's own farm where the abnormal is viewed as normal because it is seen every day (80)—may also contribute to a lack of widescale change. This barn blindness can occur at both a farm level, as well as an industry level; indeed, some practices become normalized by those working within the industry but are found abhorrent by others outside of the industry.

In further exploring the reasons for the persistence of the surplus calf challenge, framing the issue as a “wicked problem” may provide some insights. The term wicked problem was first coined by urban planners Rittel and Webber (81) as a way of describing problems which, in contrast to “tame problems,” present a unique set of challenges as a result of their inherently complex and incendiary nature. In Table 1 we show how common features of wicked problems can be related to the management of surplus dairy calves.

**Table 1:** Key features of a “wicked problem” and how aspects of current surplus dairy calf management systems could be argued to meet each of the individual features that when taken together meet the criteria for a wicked problem.

**Features assigned to wicked problems**	**Relation to surplus calf management**
• They are **difficult to clearly define** (81) and **different stakeholders have different versions of what the problem is** (82).	The challenge of surplus calf management is difficult to distill into a clear problem definition, primarily because the components of the problem are many and varied. The problem could be defined as surplus calves being slaughtered early in life and treated differently to replacement females because they are of lower economic value (as is the case in most dairy regions). But why are they of low value? Is the problem one of genetics, nutrition, husbandry, market access and demands, human perceptions of value, industry attitudes or cultural norms? Additionally, different stakeholders will place different emphases on each of the potential components of the problem.
• They are often **not stable**; the problem, constraints and evidence involved in understanding the problem (e.g., legislation, scientific evidence, resources, political alliances) are frequently evolving. They also have many **interdependencies** and are often multi-causal (82).	Evolving and interdependent influences on the management of surplus calves include market incentives/disincentives, policy, legislation, commodity price fluctuations, land availability, scientific knowledge, and evolving community attitudes/values.
• They often include **internally conflicting goals** or objectives (82).	Internally conflicting goals include the desire to achieve financially viable growth rates through accelerated/lot feeding of dairy breed calves vs. rising public opposition to concentrated animal feeding operations (83); the advantages offered by increasing use of sexed semen (70) vs. the value placed by the public on the concept of “naturalness” (84, 85); the welfare impacts of transporting calves to rearing facilities vs. at-birth euthanasia which may not compromise welfare if performed effectively but is likely to be at odds with public values.
• They have **no immediate and no ultimate test of a solution**; the full consequences of a potential solution cannot be appraised until all the waves of repercussions have completely run out (81), **and measures introduced to address the problem may lead to unforeseen consequences elsewhere** (82).	The social, environmental, and economic consequences of any changes to surplus calf management will take time to become evident. For example, increasing the number of surplus calves reared for beef or used for veal production may fail to resonate with societal values for reasons associated with production methods (e.g., cow-calf separation); proposed solutions may have a detrimental financial impact on farmers in the short term; management changes may have unforeseen impacts on the environment, land use, food security etc.
• They have **no stopping rule**, as the perfect solution will likely never be achieved (81).	Given that the socio-cultural evolution of humans is ongoing (86), the question will likely not be whether the management of surplus calves becomes “good enough” to the point that it is “solved” but rather that practices will likely require continual review in order to ensure that they align with public values in perpetuity.
• They are **socially complex**, and it is social complexity rather than the technical complexity that overwhelms most current problem-solving and project management approaches (82).	The management of surplus calves involves a diverse range of stakeholders with varying frames of reference including dairy and beef farmers, calf growers/veal producers, transporters, feedlot operators, meat processors, milk processors, wholesale, retail, food service, exporters, policy makers, compliance etc. This level of social complexity is increased again by the addition of the general public as a credible stakeholder.
• They involve **changing the behavior** and/or gaining the commitment of individual citizens (82).	Changing the status quo of surplus calf management will not only involve changing the behavior of farmers, but of all stakeholders involved along the whole supply chain (i.e., from farm to plate).

Developing a dairy industry where practices are more aligned with public values will likely be more socially sustainable (10); the question is what do these practices look like, are they economically viable, and who should be involved when discussing them?

### Addressing Complex “Wicked Problems” (The Inclusion of Voice)

Given the complex nature of surplus calf management, gaining an understanding of, and accounting for the interests of, all stakeholders and reasons that motivate conflicts of interest between them is vital (50). This will require more interactive methods of communication that can provide for democratic, interactive, and multidirectional discussion sessions (87) that stretch across different disciplines and even across public, private and civic sector organizations (88).

When addressing wicked problems, it is widely recognized that relying solely on experts and advocates is not only insufficient (89), but can actually make tackling the issues more difficult (90). As pointed out by Weary et al. (10), some solutions (see above discussion on the modified cage for hens) developed by scientists fail to gain traction with the public because (a) they do not adequately address the societal concerns that motivated the original research and, (b) they do not adequately address the perceived constraints within the industry. According to Fung (91), non-professionals may be able to contribute to the development of innovative approaches and strategies precisely because they are free from the received but obsolete wisdom of professionals and the techniques that are embedded in their organizations and their procedures.

The importance of ensuring that surplus calf management practices are not only socially acceptable but also financially viable, means that it is vital that discussions include both industry, including the farmers, their trusted advisors (e.g., veterinarians, nutritionists) and other stakeholders along the supply chain (e.g., milk processors), and the general public, in their role as both citizens and consumers, as credible stakeholders. Weary and von Keyserlingk (45) emphasize the importance of two-way conversations with the public that include not just consumers who purchase dairy products but all citizens that provide a social license for the dairy industry to operate, including those that do not consume animal products but are interested in the issues and who influence corporate and government responses. Similarly in the mining sector, it has been recognized that genuine community engagement, participation and collaborative approaches to the development of strategies to mitigate negative impacts will likely create greater community trust and acceptance in the longer term (92).

Indeed, it is becoming increasingly clear that exclusion of certain voices (i.e., the lay public), despite their lack of connection to the industry, may not be sustainable in the long term; particularly given that the younger generations are predicted to contribute significantly to the debate on choices toward new food production practices and consumption patterns (93). In contrast, the inclusion of voice from both industry as well as citizens through public participation can act as a source of “trust” and “legitimacy” (i.e., that all those involved in the conversation trust those developing potential solutions and therefore see them as legitimate) and thus can act as a means of effecting change (94).

However, the inclusion of the voice of the citizen must not be merely tokenistic; Schuppli and Fraser (95) examined factors influencing the efficacy of animal ethics review committees and found that the inclusion of community members, usually as a single or pair among a panel of several experts, often lead to them feeling outnumbered or intimidated by the expert members and their voice was often not heard. This emphasizes the importance of attempting to ensure that the inclusion of voices from various stakeholders is at least in some way representative. Despite this, Fung (91) describes the challenges associated with achieving adequate representation amongst participating voices, including: whether important interests or perspectives are excluded; whether they possess the information and competence to make good judgements and decisions; and whether participants are responsive and accountable to those who do not participate. Whilst these challenges must be acknowledged, moving to include the voice of the lay stakeholder in at least some form is an as-yet underexplored frontier when it comes to addressing wicked problems in agriculture. Using these approaches to guide and build upon traditional approaches to research, development, and extension offers a promising domain in which to break new ground.

Driving ownership of the problem and buy-in for new approaches from farmers and wider industry is also vital as any initiatives are more likely to be successful when led by producers (96) and the associated allied industries who support the agriculture industry. This buy-in for new approaches must circumvent the traditional attitudes of industry-based stakeholders who have often characterized public concerns about farm animal welfare as symptomatic of a lack of knowledge about farming and have used one-way information vehicles to educate the public (76). The challenge is to help the dairy sector as a whole to see the opportunities that change may bring as opposed to supporting a way of doing business that may become an intractable problem.

In the case of surplus calves, “fixing” the problem must go beyond refining existing practices and improving profit margins. As Bos and Koerkamp (97) state, “*the old-fashioned idea that pure technological magic will do the job, no longer applies”*. Instead, these authors argue that in order to make modern western animal production systems more sustainable, it is necessary to design systems that address multiple challenges at one time. It is not only profitability of alternative surplus calf systems that must be considered, but these types of approaches may also aid in identifying solutions for other complex issues such as animal welfare, farmer welfare, environmental impacts, and other aspects of social sustainability. Ideally, solving these issues is not done in isolation of one another as individual solutions may conflict with, or even negatively influence, the performance of other aspects of the system.

Further, when considering that human social evolution is a constant process (86), it is vital that systems for tackling complex issues such as surplus calf management are designed to accommodate and move with evolving societal values. Almost 15 years ago the Commonwealth of Australia (82) reported that any approach to tackling wicked problems will require: “*holistic, not partial or linear thinking; innovative and flexible approaches; the ability to work across agency boundaries; increasing understanding and stimulating debate on the application of the accountability framework; effectively engaging stakeholders and citizens in understanding the problem and identifying possible solutions; additional core skills such as communication and tolerating uncertainty and accepting the need for a long term focus.”* We argue that identifying a sustainable path forward regarding the issue of surplus calves produced by the dairy industry will require approaches that embrace all of these attributes. Below we discuss the use of participatory methodologies that could be used as a starting point to engage in dialogue that includes representation from industry stakeholders as well as the public.

### Examples of Participatory Methodologies

Whilst participatory methodologies vary based on who participates, they are all based on the concept that those involved co-create knowledge and make decisions together and it is their collective voice that is then linked with policy or public action (91). When it comes to the inclusion of the voice of the community, Gregory et al. (98) defines community engagement as the process of involving the community in the planning and development of policies and services by which they themselves are likely to be impacted. The three methods described in Table 2 are examples that could be used to tackle the complex surplus calf management problem and were specifically chosen since they all provide for the inclusion of voice from all sides of the issue, including the lay public, with the overall aim of identifying more meaningful, sustainable outcomes.

**Table 2:** Brief descriptions of three participatory methodologies and examples where they have been used to tackle complex problems.

**Participatory methodology**	**Description**	**Examples of methodology in use**
Deliberative forums	Deliberation is defined as the action of thinking carefully about something, especially in order to reach a decision (99). According to Gregory et al. (98), deliberative approaches to community engagement centre on involving the community in discussion and deliberation about issues, ideally leading to concrete proposals that can be adopted by policy makers. The process involves ordinary citizens being willing to tackle difficult, often value-laden problems. A key part of these types of forums is the recognition that participants will absorb educational background materials and engage in exchanges with others, who may have different perspectives, experiences, and reasons with one another and in doing so will develop their views and discover their interests (91). In contrast to the commonly-utilized focus group, Carcasson (90) emphasizes that deliberative engagement focuses on developing mutual understanding and genuine interaction across perspectives, which then provides a base to support the constant adjustment, negotiation, and creativity required to tackle wicked problems. These types of interactions do however require extensive community capacity and are indeed a cultural shift away from an over-reliance on either expert or adversarial processes.	The Irish Citizens' Assembly is an example of a deliberative forum [see Farrell et al. (100) for full description] where members of the assembly were regular citizens selected from the wider population and participated in facilitated roundtable discussions on a monthly basis. Presentations by advocacy groups and on occasions (notably when discussing abortion) personal testimonials by a number of women were also included. Together, the creation of two deliberative mini-publics in quick succession [The Irish Citizens' Assembly (2016–2018) and the early Convention on the Constitution (2012–2014)] played a significant role in supporting key referendums for constitutional change that followed [marriage equality in 2015 (101), and abortion in 2018 (102)].In a dairy-specific example, participatory policy making was recently employed in the United Kingdom to enable groups of dairy producers to deliberate and develop an antimicrobial stewardship policy [see (103) for full description]. The authors noted that “the participatory process provided comprehensive learning for all involved and allowed for the integration of science and the producers' own knowledge and experience. The process led to the development of credible and practical recommendations designed to deliver real on-farm changes” (103).
Reflexive Interactive Design (RIO)	According to Bos and Koerkamp (97), the RIO approach (a Dutch acronym for Reflexive Interactive Design) was first proposed to aid the discussions surrounding agricultural issues that are viewed to be complex and value-laden. The approach recognizes that livestock production's historical focus on volume and cost-efficiency has increasingly been confronted with a series of self-generated risks and unwanted side effects [see also (104) for discussion on risks to sustainability arising from current dairy management practices in the US]. The RIO framework places equal focus on both technical and social challenges and seeks to redesign agricultural systems in ways that can overcome these constraints to be truly sustainable (97). According to the same authors, determining the fundamental needs of all actors that are involved in a system (including farmers, the general public and consumers as well as the animals themselves) and formulating them into a “Brief of Requirements” is a key starting point of this approach. Their aim is to then redesign systems that simultaneously speak to the needs of all the different actors, instead of weighing the pros and cons of the various interests against each other (97).	An example where RIO methodology was used is the Pork Opportunities project in the Netherlands (2008-2010) [see (105)]. Briefly, the aim was to redesign the pig husbandry system to “produce pork in a way that is good for People, Planet, Profit and Pigs.” This project began with a system analysis that identified and assessed the needs of the pig, pig farmers, the environment and the consumer/citizen. Key challenges in the current pig production system were then identified as were possibilities for change. Design goals were formulated, key functions were identified and solutions to these functions were generated to fulfill the needs of all actors. A selection of these solutions was then combined to render new designs of pig husbandry systems.The RIO approach was also used by Romera et al. (106) to re-design sustainable dairy systems in New Zealand. The authors argued that this approach offered an opportunity for more profound reflexion within the dairy industry and is tailored to wicked problems and situations with apparent value conflicts. It first set out to develop desirable “ideal” systems; participants were actively encouraged to not focus on technical or economic feasibility. Only after completion of this phase were the participants then encouraged to focus on the feasibility of the concepts. Animals were considered as key actors alongside farmers as were the consumers and the New Zealand citizens; this latter aspect of the process was driven in large part by the recognition and acceptance by all involved that animals are sentient beings, whose lives could be profoundly affected by the designs if they were to be implemented.
Human Centred Design	Human Centred Design is rooted in fields such as ergonomics, computer science, and artificial intelligence (107). This approach also places priority on deeply respecting all views, recognizing that in order to develop creative, innovative solutions that are rooted in people's actual needs, the voices of all stakeholders must be included [see (108)]. The process involves three main phases: Inspiration, Ideation and Implementation, and is designed to help participants learn directly from each other, open themselves up to a breadth of creative possibilities, and then zero in on what is most desirable, feasible and viable for all actors involved [see (108)].	Human Centred Design has been used to address complex issues such as healthcare, and was utilized by The Best Babies Zone initiative, a multi-year project aimed at reducing inequities in infant mortality rates and enhancing overall population health in Oakland, California (109). As the authors describe, this approach was used to design solutions that addressed the deeply-rooted, complex social and economic conditions that are important drivers of health inequities in this region. A diverse team representing organizations from multiple sectors were invited to attend; stakeholders represented government, design, community, and economic development and individuals who worked in the neighbourhood. Collectively the goal was for all stakeholders to become familiar with the complexity of the situation in a context that deepened their understanding and empathy. Based on insights from working in the community, the team brainstormed over 100 concepts to address the design challenge and integrated community members' feedback at an early stage of the planning process.

In all three examples (see Table 2), the values and ideals of those not directly connected to an issue, but who are either affected by the issue or downstream recipients such as community members or consumers, are recognized as being of equal importance as the needs of experts or industry stakeholders in developing sustainable solutions to complex problems. It should be noted that this is in contrast to the relatively minor changes that normally follow the traditional process of getting feedback after a fundamental design had been completed by experts (that may include a representative of the humane movement (110), but not always i.e. (111) and then put forward for public comment [see process described by Canada's National Farm Animal Care Council (112)]). As Raman and Mohr (113) point out, it is not enough to simply measure social acceptance of a practice, but instead industries should aim to include all stakeholders in the co-construction of social license. Thus, engaging with all stakeholders, including the public, is a key step to ensuring that practices remain in step with evolving societal values.

Additionally, as in the case for laying hens, if the dairy industry implements solutions that fail to resonate with societal values, there is a great risk that any proposed changes may result in public disapproval as awareness of this issue grows, wasting immense resources by both the dairy industry as well as the research community. By engaging in social science research using some form of participatory methodology (see Table 2) that includes the public, we believe that the industry can minimize this challenge.

## Conclusion: The Ever-Distant Horizon

Achieving widespread adoption of socially acceptable, financially viable, and environmentally sustainable alternatives to surplus calf management is an immediate requirement to ensure the continued viability of the dairy industry. However, as complex as this specific issue is, we also recognize [as have others; (103)] that it is unrealistic to expect that the challenge of ensuring dairy animal management practices meet the needs of people, planet, profit and animals will be solved immediately and that issues hindering the sustainability of the dairy industry will be confined to the issue of surplus calves alone. There is little doubt that public scrutiny of dairy production practices will continue to increase and that this scrutiny will increasingly include challenges based on ethical grounds, including the current practice of managing surplus calves as an associated “dispensable” product of the dairy industry.

Short term measures of progress on surplus calf management will likely include improved beef market access by the dairy sector and a move away from early life slaughter. However, in addition to working on short term solutions, we encourage the industry to simultaneously begin working toward longer term solutions that will meet the future needs of the animals, the farmers who care for them, the wider agricultural sector, consumers as well as the citizens in the broader community. In doing so, related “contentious” issues such as cow-calf separation, confinement feeding (concentrated animal feeding operations), involuntary culling due to disease and lameness, and the welfare of cull cows will also need to be addressed.

The current challenge facing the global dairy industry regarding the fate of surplus calves demonstrates a clear and pressing need to engage in research that expands on the traditional focus on technical solutions by developing and evaluating participatory methodologies, enabling the dairy industry to address these ever-evolving, complex, “wicked” problems. This novel approach could potentially aid the dairy industry to clearly position itself as a leader in sustainable food production, rather than simply being reactive to issues as they arise; thereby assisting the industry in retaining its' social license to practice.

## Author's Note

The manuscript was completed as part of SB's enrollment in the University of Melbourne Dairy Residency Program.

## Author Contributions

SB and MvK both performed the literature searches and together wrote the review. All authors contributed equally to conceptualization.

## Conflict of Interest

SB is an employee of Dairy Australia where she is a Project Officer in Animal Health and Welfare. The remaining author declares that the writing of the paper was conducted in the absence of any commercial or financial relationships that could be construed as a potential conflict of interest. The handling editor declared a past collaboration with one of the authors MvK.
